# Mouse CD38-Specific Heavy Chain Antibodies Inhibit CD38 GDPR-Cyclase Activity and Mediate Cytotoxicity Against Tumor Cells

**DOI:** 10.3389/fimmu.2021.703574

**Published:** 2021-09-03

**Authors:** Natalie Baum, Marie Eggers, Julia Koenigsdorf, Stephan Menzel, Julia Hambach, Tobias Staehler, Ralf Fliegert, Frederike Kulow, Gerhard Adam, Friedrich Haag, Peter Bannas, Friedrich Koch-Nolte

**Affiliations:** ^1^Institute of Immunology, University Medical Center Hamburg-Eppendorf, Hamburg, Germany; ^2^Department of Radiology, University Medical Center Hamburg-Eppendorf, Hamburg, Germany; ^3^Mildred Scheel Cancer Career Center HaTriCS4, University Medical Center Hamburg-Eppendorf, Hamburg, Germany; ^4^Department of Biochemistry and Molecular Cell Biology, University Medical Center Hamburg-Eppendorf, Hamburg, Germany

**Keywords:** CD38, NAD^+^, antibody-dependent cellular cytotoxicity, complement-dependent cytotoxicity, multiple myeloma, nanobody, heavy chain antibody, antibody engineering

## Abstract

CD38 is the major NAD^+^-hydrolyzing ecto-enzyme in most mammals. As a type II transmembrane protein, CD38 is also a promising target for the immunotherapy of multiple myeloma (MM). Nanobodies are single immunoglobulin variable domains from heavy chain antibodies that naturally occur in camelids. Using phage display technology, we isolated 13 mouse CD38-specific nanobodies from immunized llamas and produced these as recombinant chimeric mouse IgG2a heavy chain antibodies (hcAbs). Sequence analysis assigned these hcAbs to five distinct families that bind to three non-overlapping epitopes of CD38. Members of families 4 and 5 inhibit the GDPR-cyclase activity of CD38. Members of families 2, 4 and 5 effectively induce complement-dependent cytotoxicity against CD38-expressing tumor cell lines, while all families effectively induce antibody dependent cellular cytotoxicity. Our hcAbs present unique tools to assess cytotoxicity mechanisms of CD38-specific hcAbs *in vivo* against tumor cells and potential off-target effects on normal cells expressing CD38 in syngeneic mouse tumor models, i.e. in a fully immunocompetent background.

## Introduction

NAD^+^ is released as an endogenous danger signal from cells during inflammation (1, 2). CD38, a 43 kDa type II transmembrane protein consisting of a short intracellular N-terminal domain, a transmembrane helix and a long C-terminal extracellular catalytic domain, is the major NAD^+^-hydrolyzing ecto-enzyme of mammals (3–6). NAD^+^-hydrolysis by CD38 limits the availability of NAD^+^ for extracellular-ADP-ribosyltransferases (7, 8), and generates the Ca^2+^-mobilizing metabolites ADP-ribose and cyclic ADP-ribose (9) that can be further hydrolyzed to immunosuppressive adenosine by other ecto-enzymes (10). CD38 is highly expressed in hematological malignancies including multiple myeloma (11, 12). It has been proposed that the enzymatic activity of CD38 contributes to a microenvironment favourable for tumor survival in the bone marrow niche (13, 14).

CD38 represents a promising target for monoclonal antibody (mAb)-based immunotherapy of multiple myeloma (MM) (11, 15, 16). Several CD38-specific mAbs, including daratumumab and isatuximab, have shown encouraging results in the clinic (17–20). The anti-tumor effects of these mAbs presumably reflect their ability to induce immune effector functions, such as antibody-dependent cellular cytotoxicity (ADCC) and complement-dependent cytotoxicity (CDC) (21). However, these antibodies may also induce the depletion of CD38-expressing NK cells and may have other off-target effects on normal cells expressing CD38 (22, 23). Moreover, the use of mAbs has disadvantages that include limited tissue penetration due to their large size of approximately 150 kD (24, 25).

Nanobodies are recombinant, single antigen-binding immunoglobulin variable domains (designated VHH) derived from naturally occurring camelid heavy chain antibodies (hcAbs) (26, 27). Nanobodies have several advantages over conventional antibodies, including a 10-fold smaller size (15 kDa *vs.* 150 kDa) (28, 29). To endow immune-effector functions, nanobodies can be fused to the hinge, CH2, and CH3 domains of a conventional mouse or human IgG antibody to generate nanobody-based chimeric hcAbs (30). These chimeric hcAbs lack the CH1 domain and the light chain, resulting in approximately half the molecular size of a conventional antibody (75 kDa *vs.* 150 kDa) (30).

Both, nanobodies and hcAbs are emerging as promising theranostic molecules (31–34). For example, we have recently shown that human CD38-specific hcAbs can be used to effectively target human MM cells in xenograft mouse models of systemic human lymphoma (35). Lack of reactivity with mouse CD38, however, makes it difficult to understand and assess potential off-target effects of such therapeutic antibodies on immune cells that endogenously express CD38. Substituting three amino acid residues in the CH2 domain of mouse IgG2a or human IgG1 (L234A, L235A, P329G) eliminates complement dependent cytotoxicity (CDC) as well as CD16-mediated antibody dependent cellular toxicity (ADCC) (36). These so-called LALA-PG mutants retain the thermostability and pharmacokinetics of the parental IgG (36).

We aimed to develop mouse CD38-specific nanobodies and hcAbs, to assess their binding epitopes, and to evaluate their capacity to induce cytotoxicity against tumor cells expressing CD38 *in vitro* as a basis for future *in vivo* studies of syngeneic MM models in immunocompetent mice.

## Methods

### Mice and Cells

BALB/c and C57BL/6 mice were obtained from The Jackson Laboratory or Charles River. *Cd38* ^-/-^ mice (3) were back-crossed onto the BALB/c and C57BL/6 backgrounds for 8 –12 generations. The mouse EL4 (C57BL76N lymphoma, ATCC TIB-39) and MOPC 315 (BALB/C myeloma, ATCC TIB-23) cell lines were obtained from the American Type Culture Collection. EL4 and MOPC 315 cells were cultured in RPMI-1640 medium (Gibco, Life Technologies, Paisley, UK) supplemented with 2 mM sodium pyruvate (Gibco), 2 mM L-glutamine (Gibco) and 10% (v/v) fetal calf serum (Gibco). Human HEK cells (ATCC CRL-1573) were transiently co-transfected with expression vectors for nuclear GFP and mouse CD38 (gene ID: 12494) or human CD38 (gene ID: 952) (37). The human NK-92 cell line (ACC 488) was obtained from the DSMZ German Collection of Microorganisms and Cell Cultures. NK-92 cells were stably transduced with mouse CD16 by retroviral transduction using the pSF91 retroviral vector (35). The sequence for CD16, i.e. the ectodomain of Fc*γ*RIII fused to the transmembrane and cytosolic domains of FcϵRI, was kindly provided by B. Clémenceau (Nantes, France). NK-92 cells were cultured in alpha MEM culture medium (Gibco) supplemented with 10% FCS (Gibco), 10% horse serum (Gibco), 100 IU/mL IL2 (Proleukin, Novartis) and 2 mM L-glutamine (Gibco). Primary spleen cells were obtained from wild type and *Cd38^-/-^* mice by passing spleen cell suspensions through a 70 µm cell strainer.

### Selection and Sequencing of Mouse CD38-Specific Nanobodies

Two llamas were immunized subcutaneously by ballistic cDNA immunization with an expression vector encoding the full-length open reading frame of mouse CD38. The VHH repertoire was PCR-amplified from peripheral blood lymphocytes and cloned into the pHEN2 phagemid vector as described previously (37). Selection of specific phages was performed by sequential panning of the phage library on primary splenocytes obtained from *Cd38^-/-^* and WT mice. Following extensive washing, bound phages were eluted by trypsinization. Plasmid DNA was isolated from single colonies and subjected to sequence analyses using pHEN2-specific forward and reverse primers (37).

The coding region of selected nanobodies was subcloned using NcoI/PciI and NotI into the pCSE2.5 vector (38) (kindly provided by Thomas Schirrmann, Braunschweig, Germany) upstream of either a chimeric His6x-Myc epitope tag, the coding region for the hinge and Fc domains of mouse IgG2a, or the corresponding coding region for the LALA-PG mutant (36) of mouse IgG2a (gene ID: 404711). Recombinant myc-his tagged nanobodies and chimeric nanobody-mouse IgG2c heavy chain antibodies were produced in transiently transfected HEK-6E cells (39) (kindly provided by Ives Durocher, Ottowa, Canada) cultivated in serum-free medium. Six days post transfection, supernatants were harvested and cleared by centrifugation at 4000 rpm for 10 min. Nanobodies in cell supernatants were quantified by SDS-PAGE and Coomassie staining relative to marker proteins of known quantity as described previously (37). Yields typically ranged from 0.5–3 μg Nb or hcAb per 10 µl of HEK-6E cell supernatant. Myc-His tagged nanobodies were purified by immobilized metal affinity chromatography using Ni-NTA agarose (Sigma, St Louis, MO), hcAbs by affinity chromatography on protein A immobilized on sepharose beads (GE Healthcare) (37).

### Biolayer Interferometry

The extracellular domain of mouse CD38 (aa 45–304) with intact glycosylation sites was produced as a secretory protein with a chimeric His6x-Myc epitope tag in the pCSE2.5 vector. The tagged protein was purified using immobilized metal affinity chromatography (IMAC). Affinity of hcAbs to recombinant mouse CD38 was determined by BLI-technology using a fortéBIO BLItz instrument. Assays were performed at 20°C with running buffer (PBS, 0.01% (m/v) BSA, 0.002% (v/v) Tween-20). Protein A sensors were hydrated in running buffer and loaded until saturation with hcAbs at 10 µg/ml. After washing, purified mouse CD38 (1.8 µM) was allowed to associate for 120 seconds on immobilized hcAbs, followed by dissociation for 120 seconds. Respective binding curves were referenced against antibody-loaded sensors receiving only buffer for association and dissociation steps. Curve fitting and affinity calculations were performed using Graph Pad Prism (version 7) using non-linear regression and the build-in “association then dissociation” method.

### Flow Cytometry

Purified hcAbs were conjugated *via* amino groups to Alexa Fluor^647^-fluorochrome according to the manufacturer’s instructions (Molecular Probes, Thermo Fisher Scientific). For epitope mapping analyses, EL4 cells were pre-incubated with a saturating concentration (100 nM) of unconjugated hcAbs for 30 min at 4°C, followed by addition of Alexa Fluor^647^-conjugated hcAbs (10 nM) and further incubation for 20 min at 4°C. Cells were washed and analyzed by flow cytometry on a BD-FACS Canto. Data was analyzed using the FlowJo software (Treestar). The percentage of cross-blockade was calculated from mean fluorescence intensities (MFI) as follows: (MFI in the absence of competing Abs – MFI in the presence of competing Abs): (MFI in the presence of competing Abs) x 100. Spleen cells were pre-incubated with Fc-block (BioXcell, clone 2.4G2) to minimize unspecific binding to Fc-receptors. Cells were then incubated with Alexa Fluor^647^-conjugated hcAbs, FITC-conjugated anti-B220 (BD biosciences, clone RA3-6B2), and Alexa Fluor 750 as a viability dye (ThermoFischer). Gating was performed on Alexa Fluor 750-low cells (live cells).

### Fluorometric Enzyme Assays

EL4 cells (3 x 10^5^ cells/well) were incubated at 37°C in the dark for 20 min with hcAbs (10 or 100 µg/ml) or araF-NAD (10 µM) before fluorescence measurements. After recording for 20 cycles, NGD^+^ (80 µM, Sigma, St Louis, MO) was added, followed by further incubation in the dark at 37°C. Production of cGDPR was monitored continuously for 50 min at 410 nm (emission wavelength) with the excitation wavelength set to 300 nm, using a Tecan Infinite M 200 microplate fluorimeter (37). Readings (EX300/EM410) from wells without cells were subtracted from all sample readings and values were plotted as Relative Fluorescence Units (RFU) *vs*. time. The rate of cGDPR production was calculated as the slope of the curves (RFU/s) during the linear phase of the reaction, i.e. t = 500-1200 s.

### CDC and ADCC Assays

To analyze the complement-dependent cytotoxicity (CDC) mediated by hcAbs, EL4 or MOPC 315 cells were incubated for 10 min at 4°C with hcAbs before addition of guinea pig serum (25% v/v) as a source of complement. Cells were incubated for 120 minutes at 37°C, washed and resuspended in PBS/0.2% BSA/propidium iodide before analysis by flow cytometry (35).

To analyze antibody dependent cellular cytotoxicity (ADCC) mediated by NK-92 cells, EL4 or MOPC 315 cells were incubated for 10 min at 4°C with hcAbs before addition of NK92 cells. In order to distinguish NK92 effector cells from target cells, NK92 cells were prelabelled with eFluor 450 (ThermoFisher) for 20 min at 4°C and washed three times before addition of hcAb-treated target cells at an effector to target ratio [E:T] of 3:1. Cells were co-incubated for 3 hours at 37°C, washed, and resuspended in PBS/0.2% BSA/propidium iodide before flow cytometry (35). In order to quantify cytotoxicity against target cells, gating was performed on eFluor 450-negative cells. Dead target cells were quantified using uptake of propidium iodide (PI) and decrease in forward scatter (FCS) as indicators of cell lysis. As negative controls, hcAbs carrying the ADCC and CDC abrogating LALA-PG mutations (36) were used.

### Statistical Analysis

Data were analyzed using GraphPad Prism version 7.00 (GraphPad Software). For enzyme inhibition assays, statistical significance was calculated using one-way ANOVA followed by a Bonferroni *post hoc* test for multiple comparisons. with *P*< 0.05 (*), *P* < 0.01 (**), *P* < 0.001 (***), P < 0.0001 (****). Data for CDC and ADCC assays in bar diagrams represent the mean ± SD from three independent experiments.

## Results

### Phage Display Selection Yields Five Families of Mouse CD38-Specific Nanobodies

We cloned the VHH-repertoire from blood lymphocytes of two llamas immunized (40) with a mouse CD38-encoding cDNA expression vector into M13 phage display libraries. Selection of CD38-specific phages was achieved by first panning the libraries on cells lacking CD38, i.e. splenocytes from CD38-deficient mice and YAC-1 lymphoma cells to remove unspecific binders. Libraries were then panned on cells expressing high levels of CD38, i.e. splenocytes from WT mice and EL4 thymoma cells. Sequencing of selected clones revealed 13 distinct clones (JK3, JK5, JK13, JK16, NB3, NB7, NB11, NB22, NB24, NB28, NB32, NB38, NB40 and NB42) that can be subgrouped into five distinct nanobody families based on sequence similarities in the framework and complementarity determining regions, with CDR3 lengths ranging from three to 13 amino acid residues (**Table 1**).

**Table 1:** Characteristics of mouse CD38-specific nanobodies.

Clone	Family	FR2	CDR3	length	K_D_ (nM)
**JK3**	1	QREL	YIVPYGTGSAYTV	13	> 500
**NB11**	1	QREL	YIVPYGTGSAYTS	13	423
**JK5**	2	EREF	DLFDRLVIPREST	13	102
**NB32**	3	QREV	LNY	3	147
**NB7**	4	EREF	WPPRSASWDDYDY	13	93
**NB22**	4	EREF	WPPRSASWDDYDY	13	59
**JK16**	4	EREF	WPQRSASWDDFDY	13	60
**JK13**	4	EREF	WPPRAASWDDYDY	13	113
**NB3**	4	EREF	WPPRAASWDEYDY	13	251
**NB24**	4	EREF	WPPRAANWDEYDY	13	75
**NB40**	5	QREL	DVVDDRGLGFDDY	13	19
**NB42**	5	QREL	DVVDDRGLGFDDY	13	32
**NB38**	5	QREL	DVVDSRGLGFDDY	13	33

Families were designated according to highly similar CDR3 and shared framework sequences. Variant amino acid positions in the CDR3 within a family are highlighted in grey. Affinities (K_D_) of nanobodies for the recombinant ecto-domain of CD38 were determined by biolayer interferometry.

**Figure 1:**
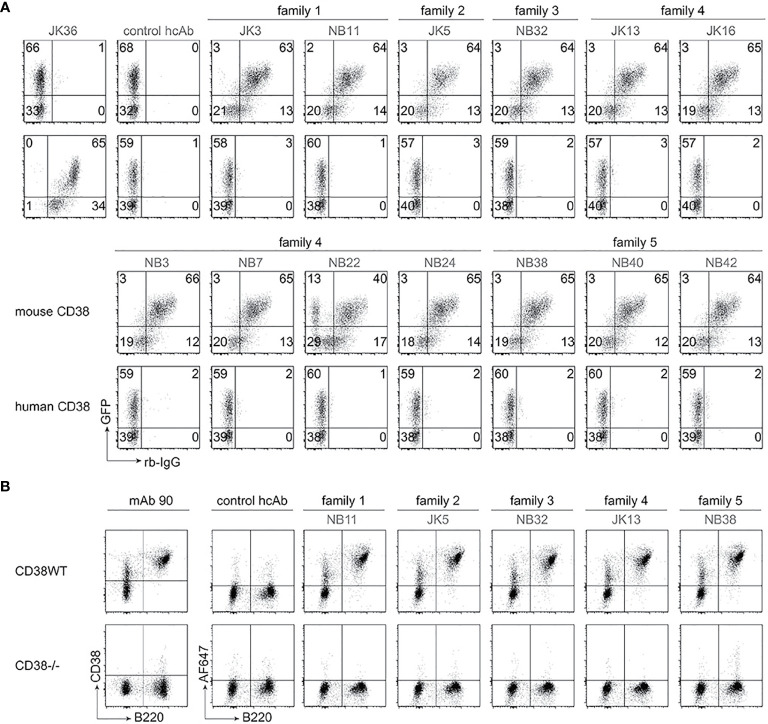
Selected heavy chain antibodies specifically recognize mouse CD38. **(A)** HEK cells were transiently co-transfected with expression constructs for GFP and either mouse CD38 (top rows) or human CD38 (bottom rows). Twenty-four hours after transfection, cells were incubated sequentially with selected nanobody-rabbit IgG hcAbs and APC-conjugated anti-rabbit IgG. Control stainings were performed with an isotype control hcAb and with human CD38-specific JK36-hcAb. Numbers indicate the percentage of cells in the respective quadrants. Data are representative of two independent experiments. **(B)** Splenocytes from wildtype (WT) and *Cd38^-/-^* mice were incubated with Alexa Fluor ^647^-conjugated hcAbs, a FITC-conjugated B220-specific mAb, and Alexa Fluor 750 as vitality dye. Control stainings were performed with an isotype control hcAb and a conventional mouse CD38-specific mAb (clone 90). Gating was performed on live (Alexa Fluor 750-low) cells. Data are representative of two independent experiments.

In order to generate nanobody-based mouse heavy chain antibodies (hcAbs), the VHH-coding region was fused to the hinge, CH2 and CH3 domains of mouse IgG2a. These hcAbs were produced as secreted recombinant proteins in transiently transfected HEK cells grown in serum-free medium. Affinities of the hcAbs were determined by biolayer interferometry using the recombinant ecto-domain of mouse CD38 (**Table 1**). The results reveal moderate affinities in the two and three digit nanomolar range; family 1 nanobodies had the lowest affinities (> 400 nM), family 5 nanobodies the highest affinities (19-33 nM). In order to verify the specificity of the selected antibodies, we performed flow cytometry analyses of HEK cells co-transfected with GFP and either mouse CD38 or human CD38 (**Figure 1A**). The results show specific binding of the selected hcAbs to mouse CD38 but not to human CD38. Flow cytometry analyses of splenocytes obtained from wild type and from CD38-deficient mice confirmed the specific binding of all hcAbs to native mouse CD38 (**Figure 1B**).

### Cross Blockade Analyses Reveal Binding of Nanobody-Based hcAbs to Three Non-Overlapping Epitopes of Mouse CD38

Next, we aimed to assess whether the identified anti-mouse CD38-specific hcAbs recognize overlapping or distinct epitopes on mouse CD38. To this end, we performed cross blockade flow cytometry analyses. We monitored the binding of Alexa Fluor^647^-conjugated hcAbs (JK3-hcAb, JK5-hcAb, JK13-hcAb, JK16-hcAb) to EL4 thymoma cells in the presence of excess unlabelled hcAbs (**Table 2**). The results show that the selected hcAbs fall into three distinct binding groups. Group 1 hcAbs block binding of hcAbs derived from nanobodies JK3 and NB11 (both family 1). These nanobodies recognize an overlapping epitope designated *epitope 1*. Group 2 hcAbs block binding of hcAbs derived from nanobodies JK5 (family 2) and NB32 (family 3). These nanobodies recognize an overlapping epitope designated *epitope 2*. Group 3 hcAbs block binding of hcAbs derived from nanobodies JK13, JK16, NB3, NB7, NB22, NB24 (family 4) and NB38, NB40, and NB42 (family 5). These nanobodies recognize an overlapping epitope designated *epitope 3*. Remarkably NB32-hcAb (epitope 2), which has the shortest CDR3, seemed to enhance binding of hcAbs that bind to the epitope 1 (JK3-hcAb) and epitope 3 (JK13-hcAb, JK16-hcAb). Note that JK13-hcAb and JK16-hcAb (both epitope 3) blocked binding of JK3-hcAb (epitope 1), suggesting that these hcAbs either sterically interfere with binding of JK3-hcAb or alter the conformation of mouse CD38 so as to inhibit binding of JK3-hcAb.

**Table 2:** Epitope mapping of nanobody-based mouse CD38-specific hcAbs.

ep	Fam	Nb	JK3^647^	JK5^647^	JK13^647^	JK16^647^
**1**	1	JK3	98	17	27	18
**1**	1	NB11	74	5	17	29
**2**	2	JK5	29	97	33	5
**2**	3	NB32	-58	82	-22	-8
**3**	4	JK13	55	-5	99	100
**3**	4	JK16	79	25	95	98
**3**	4	NB3	-19	4	72	78
**3**	4	NB7	-22	0	71	82
**3**	4	NB22	-5	3	66	65
**3**	4	NB24	-27	0	75	79
**3**	5	NB38	6	15	79	83
**3**	5	NB40	0	14	75	77
**3**	5	NB42	12	13	76	75

EL4 thymoma cells were incubated for 30 min at 4 °C with unconjugated hcAbs (indicated on the left) before addition of Alexa Fluor^647^-conjugated hcAbs (indicated on top). Cells were further incubated for 30 min at 4 °C, washed twice and analyzed by flow cytometry. Numbers indicate the percentage maximal blockade of the mean fluorescence intensity of cells labelled in the presence of competing hcAbs. Negative numbers indicate enhanced labelling of cells in the presence of the competing hcAbs. Efficiency of inhibition is indicated by different shades of grey (dark grey: > 80% inhibition, light grey: 50–80% inhibition). Self-blockade by the nanobody used for labelling is indicated by highlighted boxes in the diagonal. HcAbs that blocked binding of each other were assigned to the same epitope.

### Nanobody-Based hcAbs of Families 4 and 5 Inhibit the GDPR Cyclase Activity of Mouse CD38

**Figure 2:**
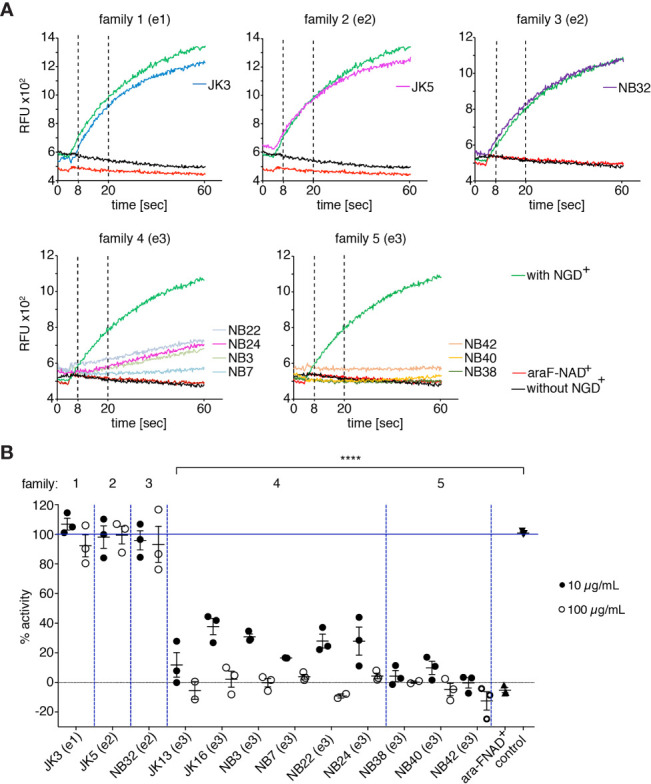
hcAbs of families 4 and 5 inhibit the GDPR-cyclase activity of mouse CD38. EL4 thymoma cells were incubated with the indicated hcAbs or 10 μM araF-NAD^+^ for 15 min at 37°C before fluorescence measurements with a microplate reader. After the first 20 cycles, 50 μM NGD^+^ was added and kinetic fluorescence reading (ex/em: 300/410 nm) was continued for 60 min. **(A)** Representative fluorimetry plots for cells incubated with the indicated hcAbs. Controls included cells incubated with (green) or without (black) NGD^+^, and cells incubated with NGD^+^ and araF-NAD^+^ (red). Vertical dotted lines at 8 and 20 min depict the time points used for calculation of slopes depicted in **(B)**. **(B)** Each dot indicates the slope of the curve during the linear phase (t = 8-20 min), relative to the slope of the curve obtained from control cells incubated with NGD^+^ alone (n = 3). Statistical significance was calculated using one-way ANOVA followed by a Bonferroni *post hoc* test for multiple comparisons. ****p < 0.0001. Data are representative of three independent experiments.

To analyze the potential functional effects of the selected hcAbs on the enzyme activity of mouse CD38 on cells, we employed a fluorometric GDPR cyclase assay (37, 41–43). This assay uses NGD^+^ (which carries a guanine nucleobase instead of adenine) instead of NAD^+^ as substrate. We incubated CD38-positive EL4 cells with either CD38-specific hcAbs for 15 min before addition of NGD^+^ and monitored the increased fluorescence of the product cGDPR by fluorimetry (44) (**Figure 2**). The results showed a continuous increase of cGDPR during incubation of EL4 cells with NGD^+^ in the absence of antibodies. As a control, we used the NAD^+^-analogue nicotinamide 2-deoxy-2-fluoroarabinoside adenine dinucleotide (araF-NAD^+^), a highly specific inhibitor of mouse CD38 (7, 45). Addition of araF-NAD^+^ effectively abrogated the increase of cGDPR, indicating that the increased fluorescence is largely due to CD38 on the surface of EL4 cells. Addition of hcAb from family 1 (JK3-hcAb), family 2 (JK5-hcAb), and family 3 (NB32-hcAb) had little if any effect on the enzyme activity of EL4 cells (**Figures 2A–C**). Addition of family 4 hcAbs (hcAbs NB3, NB7, NB22, NB24) showed significant, but varying levels of enzyme inhibition (**Figure 2D**). The most potent inhibitory effect of the GDPR cyclase activity was observed for all tested hcAbs from family 5 (hcAbs NB38, NB40, NB42) (**Figure 2E**). **Figure 2F** allows for direct visual comparison of the inhibitory effects of all tested hcAbs and demonstrates the dose dependency of the hcAbs used.

### All CD38-Specific hcAbs Mediate Effective ADCC

**Figure 3:**
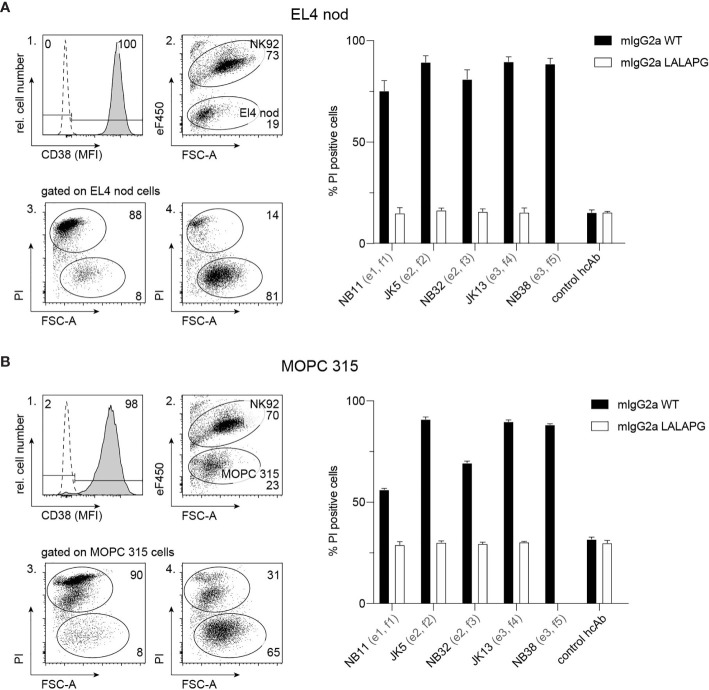
hcAbs of all five families effectively induce antibody-dependent cellular cytotoxicity against CD38-expressing thymoma and myeloma cell lines. Cell surface expression of CD38 by EL4 thymoma **(A)** and MOPC 315 myeloma **(B)** cells was assessed by flow cytometry using a fluorochrome-conjugated mouse CD38-specific mAb and an isotype control (open histogram) (panel 1). To assess the capacity of hcAbs to induce ADCC, EL4 cells **(A)** or MOPC 315 cells **(B)** were co-cultured with eFluor 450-labelled NK-92 cells for 3h at 37°C at an effector to target ratio of 3:1 in the presence of CD38-specific mouse IgG2a hcAbs from all five nanobody families. As controls, we used the same mouse IgG2a hcAbs carrying the LALA-PG mutation that abrogate FcR-binding and a non-binding control hcAb. Cells were then incubated with propidium iodide (PI) and analyzed by flow cytometry. Representative dot plots illustrate the clear separation of eFluor 450-postive effector cells and eFluor 450-negative target cells (panel 2). To assess cytotoxicity against target cells, gating was performed on eFluor 450-negative cells (panels 3, 4). Representative dot plots in panels 3 and 4 illustrate the clear distinction of dead target cells (PI-positive, low forward scatter/FSC-A) from live target cells (PI-negative, FSC^high^). Panel 3 shows the results of cells incubated with JK5 hcAb, panel 4 shows the results of cells incubated with the isotype control hcAb. Numbers in panels 2-4 indicate the percentage of cells in the indicated gated populations. Epitopes and nanobody families are indicated in parentheses behind the nanobody names. Data in bar diagrams represent mean ± SD from three independent experiments.

To analyze the capacity of mouse CD38-specific hcAbs to induce ADCC, EL4 thymoma cells or MOPC 315 myeloma cells were used as target cells and NK-92 cells stably transfected with mouse Fc-receptor III (CD16) were used as effector cells. Flow cytometry analyses confirmed high expression of CD38 by EL4 and MOPC 315 cells (**Figures 3 A, B**, panel 1), while human NK-92 cells were negative for mouse CD38 (not shown). To monitor ADCC, EL4 and MOPC 315 cells were incubated with eFluor 450-labelled NK92 cells in the absence or presence of Nb-based mouse IgG2a hcAbs. To evaluate cell death, we monitored uptake of propidium iodide and decrease in forward scatter by flow cytometry (**Figures 3 A, B**, panels 2–4). The results show that all analyzed hcAbs mediated effective killing of EL4 and MOPC 315 target cells. In contrast, only background levels of cell death were observed in the presence of mouse IgG2a hcAbs carrying the three LALA-PG mutations that abrogate binding to Fc-receptors (36).

### CD38-Specific hcAbs of Families 2, 4, and 5 Mediate CDC

**Figure 4:**
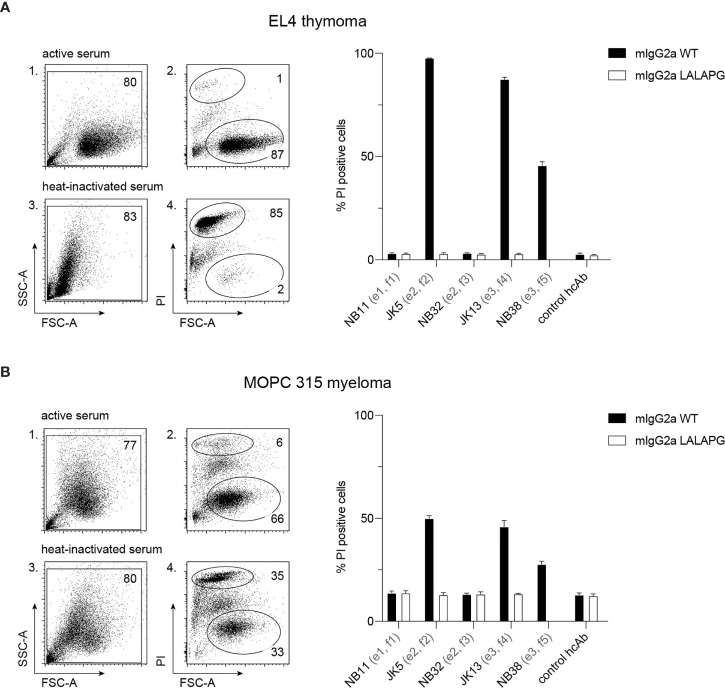
hcAbs of families 2, 4, and 5 mediate complement-dependent cytotoxicity of cells against CD38-expressing thymoma and myeloma cell lines. EL4 thymoma **(A)** and MOPC 315 myeloma **(B)** cells were incubated for 120 min at 37°C with the indicated CD38-specific mouse IgG2a hcAbs in the presence of 25% guinea pig serum as a source of complement. The same serum was pretreated for 10 min at 70°C to inactivate complement components and used as a control (heat-inactivated serum). As additional controls, we used LALA-PG mutant hcAbs (with abrogated C1q-binding) and a non-binding control hcAb. Cells were stained with propidium iodide and analyzed by flow cytometry to quantify the percentage of dead (PI-positive, FSC^low^) cells. Gating was performed to exclude cellular debris with very low FSC and low SSC. Numbers in panels 1-4 indicate the percentage of cells in the indicated gated populations. Data in the bar diagrams represent mean ± SD from three independent experiments.

To analyze the potential of hcAbs to induce CDC, we incubated EL4 and MOPC 315 cells with saturating amounts of mouse IgG2a hcAbs and guinea pig serum as a source of complement. As indicators of cell lysis, we monitored permeabilization of cells to the DNA-staining dye propidium iodide and decrease in forward scatter using flow cytometry (**Figure 4**). The results show that hcAbs JK5, JK13, and NB38 from families 2, 4 and 5 mediate effective CDC of EL4 and MOPC 315 target cells. In contrast, only background levels of cell death were observed when incubations were performed in the presence of the LALA-PG mutants of the corresponding hcAbs that abrogate binding of complement factor C1q (36).

## Discussion

From immunized llamas, we selected 13 mouse CD38-specific hcAbs that derive from five nanobody families, each of which carries a common framework region and a highly similar CDR3. These hcAbs bind specifically to three distinct epitopes of murine CD38.

All epitope 3-directed hcAbs (i.e., families 4 and 5) inhibited the GDPR-cyclase activity in a dose dependent fashion. In contrast, hcAbs of families 1, 2 and 3 had little if any effect on the GDPR-cyclase activity. This NGD^+^-based assay is commonly used to assess the effect of antibodies on CD38 enzyme activity (37, 43, 46). A limitation of this assay is that it only allows an estimate of the allosteric inhibitory effect of antibodies on the GDPR-cyclase, but not necessarily on the ADPR-cyclase or NAD-glycohydrolase activities of CD38. It is possible that nanobodies might affect cyclase and NADase activities differentially. Indeed, in a recent study we found that both, daratumumab and human CD38-specific hcAb 1067, inhibited the GDPR-cyclase and ADPR-cyclase activities of human CD38 (47), while neither daratumumab nor hcAb 1067 had any detectable effect on the NADase activity of CD38. It has been proposed that CD38 contributes to shaping an immunosuppressive tumor microenvironment (TME) by fuelling the conversion of NAD^+^ to immunosuppressive adenosine (14, 48–50). Since inhibition of the NADase activity of CD38 is more relevant in this context than inhibition of its cyclase activities, there remains a need for better CD38-inhibitory antibodies.

The CD38-specific hcAbs from all five families, irrespective of their binding epitopes induced potent ADCC of murine cell lines. This in line with the results of our previous studies showing that human CD38-specific nanobody-based hcAbs potently induced ADCC of several human lymphoma and myeloma cell lines, including LP-1 myeloma, CA-46 and Daudi Burkitt lymphoma (35, 51).

Members of hcAbs families 2, 4 and 5, but not of families 1 and 3 also mediated CDC. These findings differ from those of our previous studies with hcAbs directed against human CD38, in which we found that the 22 nanobody-based hcAbs directed against 3 different epitopes of human CD38 showed little if any capacity to induce CDC against different human CD38 expressing lymphoma cell lines (35, 51). Similar findings were reported for a panel of 42 human mAbs, of which only a single Ab, daratumumab, was able to induce CDC (35, 52).

The ability of daratumumab to induce CDC was strongly potentiated by point mutations of residue E345 or E430 that facilitate oligomerization of CD38-bound antibodies into ordered hexamers on the cell surface (53, 54). Similarly introduction of the E345R mutation also markedly enhanced the CDC potency of human CD38-specific hcAbs (51).

New structural insights regarding the CDC-inducing potency have recently been obtained for antibodies directed against the B-cell membrane protein CD20 (54). These Abs have been subdivided into two groups, which either recruit complement effectively (type I) or not (type II). A cryo-electron microscopy analysis of the Fab fragments of such antibodies in complex with full length dimeric CD20 found that the CD20 dimer bound only one Fab arm of the type II mAb obinutuzumab, but two Fab fragments of the type I mAbs rituximab and ofatumumab (55). These findings indicate that type I antibodies act as molecular seeds that allow formation of oligomeric complexes, while type II antibodies preclude recruitment of additional complexes. Since CD38 can also form dimers and oligomers (6, 56), it is tempting to speculate that mouse CD38-specific hcAbs of families 2, 4, and 5 can similarly act as molecular seeds that facilitate formation of oligomeric complexes of CD38 on the cell surface and thereby enhance CDC.

*In vivo* studies are ultimately needed to assess the potential therapeutic efficacy of the hcAbs reported here in mouse myeloma models. Of note, we have previously shown that nanobody-based hcAbs can achieve therapeutic efficacy *in vivo* in xenograft mouse models using our previously generated hcAbs directed against human CD38 (35). These human CD38-specific hcAbs reduced the growth of a systemic lymphoma and prolonged the survival of tumor bearing SCID mice. The specificity for murine CD38 makes our hcAbs unique tools to simultaneously assess the cytotoxicity mechanisms of CD38-specific hcAbs *in vivo* against tumor cells and their potential off-target effects on normal cells expressing CD38 in syngeneic mouse tumor models, i.e. in a fully immunocompetent background.

## Data Availability Statement

The raw data supporting the conclusions of this article will be made available by the authors, without undue reservation, to any qualified researcher.

## Ethics Statement

The animal study was reviewed and approved by the animal welfare commission (Amt für Verbraucherschutz, Lebensmittelsicherheit und Veterinärwesen Hamburg, Nr. A8a/694).

## Author Contributions

PB and FK-N conceived the project. All authors established experimental procedures. NB, PB, and FK-N wrote the manuscript. All authors contributed to the article and approved the submitted version.

## Funding

Supported by grants SFB1328-A05 and Z02 from the Deutsche Forschungsgemeinschaft to RF, FH, and FK-N and by a grant from the Hamburger Krebsgesellschaft to ME.

## Conflict of Interest

FH and FKN receive a share of antibody sales via MediGate GmbH, a wholly owned subsidiary of the University Medical Center Hamburg-Eppendorf. PB and FKN are coinventors on a patent application on CD38-specific nanobodies.

The remaining authors declare that the research was conducted in the absence of any commercial or financial relationships that could be construed as a potential conflict of interest.

## Publisher’s Note

All claims expressed in this article are solely those of the authors and do not necessarily represent those of their affiliated organizations, or those of the publisher, the editors and the reviewers. Any product that may be evaluated in this article, or claim that may be made by its manufacturer, is not guaranteed or endorsed by the publisher.

## Abbreviations

ADCC, antibody dependent cellular cytotoxicity; CDC, complement-dependent cytotoxicity; CDR, complementarity determining region; Fc, crystallizing fragment; hcAb, heavy chain antibody, Ig immunoglobulin; kDa, kilodalton; MM, multiple myeloma; NAD^+^, nicotinamide adenine dinucleotide; mAb, monoclonal antibody; Nb, nanobody; VHH, variable domain of a camelid heavy chain antibody.
